# Sensitivity Distribution Properties of a Phase-Shifted Fiber Bragg Grating Sensor to Ultrasonic Waves

**DOI:** 10.3390/s140101094

**Published:** 2014-01-09

**Authors:** Qi Wu, Yoji Okabe, Kazuya Saito, Fengming Yu

**Affiliations:** Institute of Industrial Science, University of Tokyo, 4-6-1 Komaba, Meguro-ku, Tokyo 153-8505, Japan; E-Mails: okabey@iis.u-tokyo.ac.jp (Y.O.); saito-k@iis.u-tokyo.ac.jp (K.S.); houmei@iis.u-tokyo.ac.jp (F.Y.)

**Keywords:** optical fiber sensor, phase-shifted fiber Bragg grating, ultrasonic detection

## Abstract

In this research, the sensitivity distribution properties of a phase-shifted fiber Bragg grating (PS-FBG) to ultrasonic waves were investigated employing the surface attachment method. A careful consideration was taken and examined by experimental results to explain that the distances and angles between the sensor and ultrasonic source influence not only the amplitudes, but also the initial phases, waveforms, and spectra of detected signals. Furthermore, factors, including the attachment method and the material's geometric dimensions, were also discussed. Although these results were obtained based on PS-FBG, they are also applicable to a normal FBG sensor or even an optical fiber sensor, due to the identical physical changes induced by ultrasonic waves in all three. Thus, these results are useful for applications of optical fiber sensors in non-destructive testing and structural health monitoring.

## 1. Introduction

Many researchers have investigated the use of a fiber Bragg grating (FBG) as an effective ultrasonic sensor due to this grating's inherent advantages, including its flexibility, immunity to electromagnetic interference, corrosion resistance, small size, and ability to be embedded into various materials. In these areas, an FBG outperforms the traditional lead-zirconate-titanate (PZT) sensor. The applications of ultrasonic detection by FBG have two main features. Some researchers use the FBG as a hydrophone, which involves immersing the FBG in water or another liquid [1]. In this case, the ultrasonic frequency is typically high, and the fiber is under isotropic stress. Alternately, other researchers use FBGs in the field of non-destructive testing (NDT) or structural health monitoring (SHM), where the FBG is normally attached to material surfaces or embedded into materials [2,3]. In these cases, the utilized frequency range is normally under 2 MHz, and stress is always exerted in a dominant direction. While, in the first case Rosenthal *et al.* have delved into the spatial characterization of optical fiber as a hydrophone [4], the sensitivity distribution properties of optical fibers in the second case have not been studied in detail.

Recently, in order to resolve the conflict between bandwidth and sensitivity in normal ultrasonic FBG sensors, phase-shifted fiber Bragg gratings (PS-FBG) were introduced by the authors and other researchers as a potential alternative to ultrasonic sensors [5,6]. The effective grating length of the PS-FBG sensor is much shorter than its grating length, leading to better responses to high-frequency ultrasonic waves. Additionally, the steep slope in the peak area of PS-FBG effectively improves the sensitivity of the sensing system, because the sensitivity of the demodulation system based on laser-source positively relates to the slope of the spectrum according to [7]. For example, the authors have successfully used PS-FBG in the acousto-ultrasonic [5] and the acoustic emission (AE) methods [8]. In these experiments, the PS-FBG sensor was glued in line to the ultrasonic source, which is a setup similar to other experiments where normal FBGs were used. However, a network with many FBG or PS-FBG sensors is necessary in both the acousto-ultrasonic and the AE method. Thus, to understand the sensitivity distribution properties of FBG or PS-FBG, it is important to optimize the sensor network for NDT and SHM. Furthermore, signal analysis to determine the damage types or identify the impact position or AE positions also demands systematic study in this field.

Although, compared to a normal FBG, a PS-FBG has broader bandwidth and higher sensitivity to ultrasonic waves, the theory of ultrasonic detection for PS-FBG is identical because, under the influence of ultrasonic waves, PS-FBGs undergo the same physical changes as optical fibers. Once we have obtained the sensitivity distribution properties of the PS-FBG, we could also deduce these results for normal fiber sensors in more common cases. The sensitivity distribution properties of an optical fiber sensor are mainly determined by the distance from and the angle to the ultrasonic source, but the distribution is also influenced by many other factors, such as effective sensing length and cladding property. Furthermore, when the fiber is attached to the surface of a material, additional factors should be considered, including coupling performance, the ultrasonic waves' properties, and the material's geometric structure. In common cases of NDT or SHM, the target material has a plate shape, while the propagating ultrasonic wave is a Lamb wave. Thus, in this study, we comprehensively researched the sensitivity distribution properties of PS-FBG when the sensor was attached to the surface of an aluminum plate. The structure of this paper is as follows: firstly, the experimental setup is introduced; then, theory analysis is explained; and lastly, the results are shown and discussed.

## 2. Experimental Setup

**Figure 1.**
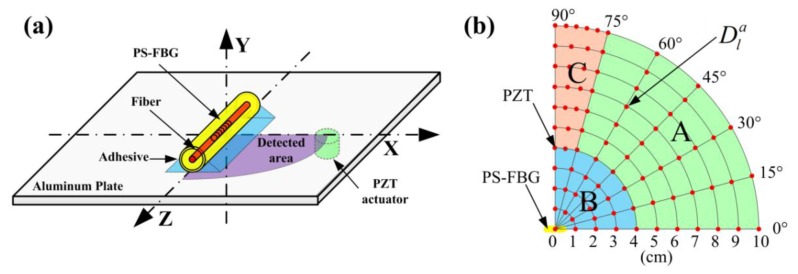
Schematic diagram of experimental setup. (**a**) Acousto-ultrasonic method was used to research the sensitivity distribution properties of a PS-FBG sensor on an aluminum plate. (**b**) Data were measured on 82 different dots distributed in a quarter-circle range.

Figure 1 is a schematic diagram of the experimental setup based on the acousto-ultrasonic method. A PS-FBG manufactured by Fujikura Company (Tokyo, Japan) with a grating length of 5 mm and a diameter of 150 μm was used as an ultrasonic sensor. Using cyanoacrylate adhesive, the PS-FBG was glued to an aluminum plate with dimensions of 50 × 50 × 0.3 (L × W × H) cm^3^. The size of the plate was large enough that the waveform had only one envelop in the detection time interval of 80 μs because reflected waves do not exist in this time interval.

The Bragg wavelength shift caused by the strain from the ultrasonic wave was demodulated by the balanced sensing technique [7]. By adjusting the wavelength of tunable laser source to the 3 dB position of the peak area of PS-FBG carefully, the balanced photo-detector can remove the DC voltage, double the AC voltage while remove the laser intensity noise which is the mainly noise source. Thus, this technique has a very low noise level, and the output electrical voltage is linearly proportional to the Bragg wavelength shift. Therefore, this technique can describe the Bragg wavelength shift correctly and precisely.

Serving as a point-like ultrasonic source, a PZT ultrasonic actuator (M31, Fuji Ceramics, Fujinomiya, Japan) with a diameter of 3 mm was driven by an electrical pulse with a peak-to-peak voltage of 75 V. The input signal was a one-cycle sinusoidal wave at 400 kHz with a Hamming window, and thus the corresponding frequency range reached approximately 1 MHz to simulate AE signals with broad bandwidth.

Using a high-acoustic-impedance ultrasonic couplant, the PZT actuator was glued to 82 different excitation dots on the aluminum plate's bottom surface. These dots were distributed from 0 to 10 cm and from 0° to 90° in a quarter-circle range, as shown in Figure 1b. To ensure careful observation of the waveform's change, the distribution of the dots from 75° to 90° was denser than the distribution in other areas. Because the amplitudes of detected waveforms were greatly affected by the attachment condition, data were collected by repeating the measurement three times to guarantee the reliability of the experimental results.

For convenient discussion, three naming rules were introduced. Firstly, a Cartesian coordinate system was established on the plate in which the phase-shifted area of the PS-FBG was set as the origin and the axial direction of the fiber was set as the Z-axis, as shown in Figure 1a. Then, the excitation dots were designated $D_{1}^{a}$. The superscript *a* and the subscript *l* denote the angle and length between the actuator and the sensor, respectively. Finally, because of different observational phenomena present in this experiment, the excitation area can be roughly divided into three parts, marked as A, B, and C, as shown in Figure 1b.

## 3. Theoretical Analysis

### 3.1. Bragg Wavelength Shift by Strains in Three Orthogonal Axes

**Figure 2.**
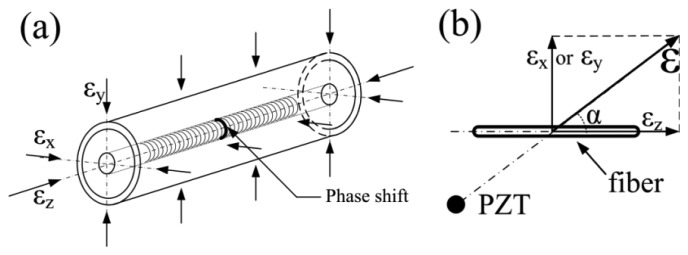
Theoretical principle. (**a**) PS-FBG subjected to strains in three orthogonal axes. (**b**) Directional strain impacts on an optical fiber.

A FBG is manufactured by writing a periodic variation of refractive index along a certain length of single-mode optical fiber via ultraviolet light. When a π phase shift is inserted into the middle of the grating area, a PS-FBG can be manufactured, as shown in Figure 2a. Although the equations to describe the spectra of PS-FBG and normal FBG differ, the Bragg wavelength expression for both gratings can be described simply by Equation (1) [9]:

(1) $$\lambda_{B}=2n\Lambda$$

where *λ_B_* is the Bragg wavelength, *n* is the average refractive index, and Λ is the average grating period.

Firstly, we consider an ideal case; *i.e.*, the fiber is in a free space. Furthermore, the strain from ultrasonic wave perfectly couples to the fiber, and the wavelength of the ultrasonic wave is much longer than the grating length. In this case, the *n* and Λ change according to Equations (2) and (3), respectively [10]:

(2) $$n=n_{0}-\frac{n_{0}^{3}}{2}\left[P_{12}{ɛ}_{z}+\left(P_{11}+P_{12}\right)\frac{{ɛ}_{x}+{ɛ}_{y}}{2}\right]$$

(3) $$\Lambda =\left(1+{ɛ}_{z}\right)\Lambda_{0}$$

where *n_0_* is the initial average refractive index, Λ_0_ is the initial average grating period, *P_11_* and *P_12_* are Pockel's strain-optic coefficients, and *ε_x_*, *ε_y_*, and *ε_z_*, are the strains along the three orthogonal axes X, Y, and Z, respectively.

Herein, we consider two different conditions. In the first case, the ultrasonic wave only propagates along the Z-axis. Because the fiber approximates isotropic material, the lateral strains are related to the axial strain by a standard Poisson ratio *ν*. Thus, in this case, the strains in the three axes have the relations expressed in Equation (4):

(4) $${ɛ}_{x}={ɛ}_{y}=-\nu {ɛ}_{z}$$

By substituting Equation (4) into Equations (1)–(3), the Bragg wavelength shift Δ *λ_B_* is expressed in Equation (5):

(5) $$\begin{array}{l} \Delta \lambda_{B}=C_{z}\lambda_{B}{ɛ}_{z}\\ C_{z}=1-\frac{n_{0}^{2}}{2}\left[P_{12}-\nu \left(P_{11}+P_{12}\right)\right] \end{array}$$

Alternately, when the fiber only experiences lateral strain along the X-axis or Y-axis, the stains in the three orthogonal axes have the relations shown in Equation (6). These relations result because the cylindrical fiber is rotationally symmetric in the X- and Y-axes, and the fiber material is quasi-isotropic:

(6) $$-\nu {ɛ}_{x}={ɛ}_{y}={ɛ}_{z}\;\text{or}\;{ɛ}_{x}=-\nu {ɛ}_{y}={ɛ}_{z}$$

By substituting Equation (6) into Equations (1)–(3), the Bragg wavelength shift Δ *λ_B_* is expressed in Equation (7):

(7) $$\begin{array}{l} \Delta \lambda_{B}=C_{i}\lambda_{B}{ɛ}_{i}\\ C_{i}=-\nu -\frac{n_{0}^{2}}{2}\left[-\nu P_{12}+\frac{1-\nu }{2}\left(P_{11}+P_{12}\right)\right] \end{array}$$

where *i* = *x* or *y*. In a standard single-mode fiber, *n_0_*, *ν*, *P_11_* and *P_12_*, have values of about 1.4453, 0.17, 0.121, and 0.27, respectively. Thus, *C_x_* = *C_y_* = −0.29150, and *C_z_* = 0.7874.

In the first case, the Bragg wavelength shift is proportional to the stain in the Z-axis, according to Equation (5), and in the second case, the shift is proportional to the strain in the X- or Y-axis, according to Equation (7). The absolute value of *C_z_* is larger than the absolute value of *C_x_* or *C_y_*. However, the negative sign in *C_x_* and *C_y_* means that the same strain will cause the Bragg wavelength to shift in the opposite direction, reflected as an opposite initial phase in detected waveforms.

### 3.2. Influence from Surface Attachment Method

The above analysis is based on the assumption that the ultrasonic wave perfectively couples to the optical fiber. However, in both our experimental condition and other practical conditions where the fiber is attached to the surface of a plate, the effective ultrasonic coupling performance between fiber and plate should be considered. Thus, we add the influence from the surface attachment method. The strain from the Z-axis has the best coupling performance, while the strain from the X-axis is difficult to couple to the fiber due to the limited effective contact area. Although no strain exists from the Y-axis because of the fiber's surface attachment method, acceleration in the Y-axis will cause dynamic strain to influence the fiber. Thus, in the actual case, the parameters *C_x_*, *C_y_*, and *C_z_* will deteriorate to *C_x_*, *C_y_*, and *C_z_*, the absolute values of which have relations |*C_z_*| > |*C_x_*| and |*C_z_*| > |*C_y_*|. The exact actual values differ depending largely on coupling performance; however, the negative sign in *C_x_* and *C_y_* will always exist.

### 3.3. Bragg Wavelength Shift by Directional Strain

A more common case in which the ultrasonic wave propagates from a direction other than the three orthogonal axes is considered. When the PZT actuator and the PS-FBG sensor have an angle of *α*, as shown in Figure 2b, the actual strain applied to the fiber can be written as the composite of the strains from the Z-axis and the strain from the X- or Y-axis. For example, when the stain is in the X-Z plane, the Bragg wavelength shift under strain *ε* can be written as:

(8) $$\Delta \lambda_{B}=c_{x}\lambda_{B}{ɛ}_{x}+c_{z}\lambda_{B}{ɛ}_{z}=\left(c_{x}\sin\alpha +c_{z}\cos\alpha \right)\lambda_{B}ɛ$$

Due to the negative sign in *C_x_*, there should exist an angle *α_min_* where the Bragg wavelength shift is minimal. Because the Bragg wavelength shift is proportional to the output voltage, *α_min_* can be predicted according to Equation (9), where *V*|*ε_z_* and *V*|*ε_x_* are the amplitudes of the detected waveforms measured when the stain exists only in the Z-axis and the X-axis, respectively:

(9) $$\alpha_{\min}=\tan^{-1}\left(-\frac{c_{z}}{c_{x}}\right)=\tan^{-1}\left(-\frac{{\Delta \lambda_{B}|}_{{ɛ}_{x}=0}}{{\Delta \lambda_{B}|}_{{ɛ}_{z}=0}}\right)=\tan^{-1}\left(-\frac{V|{ɛ}_{z}}{V|{ɛ}_{x}}\right)$$

### 3.4. Frequency Responses to Different Angles

In this part of analysis, we add consideration of the influence from the wavelength of ultrasonic waves because ultrasonic wave changes the grating periodically rather than uniformly as quasi-static strain. As demonstrated in previous research [11,12], the fiber's frequency response to ultrasonic waves depends on the effective sensing length. In general, as the effective sensing length lengthens, the high-frequency response worsens. When an ultrasonic wave propagates along the Z-axis, the effective sensing length of PS-FBG is the effective grating length with about a few hundred micrometers [6]. When an ultrasonic wave propagates along the X- or Y-axis, the effective sensing length is the fiber's 150-micrometer diameter, which is shorter than the effective grating length. Thus, without considering the influence from distance or the surface attachment method, the PS-FBG sensor will have a higher response to the ultrasonic wave that propagates in the X- or Y-axis. Furthermore, considering the actual case, the sensitivity decrement of PS-FBG to high frequencies is slower than the decrement to low frequencies when the angle between sensor and actuator changes from 0° to 90°. Paralleling this effect, the detected signal cannot be completely removed at *α_min_* because the exact *α_min_* differs depending on frequency.

## 4. Results

### 4.1. Sensitivity Distribution on an Aluminum Plate

**Figure 3.**
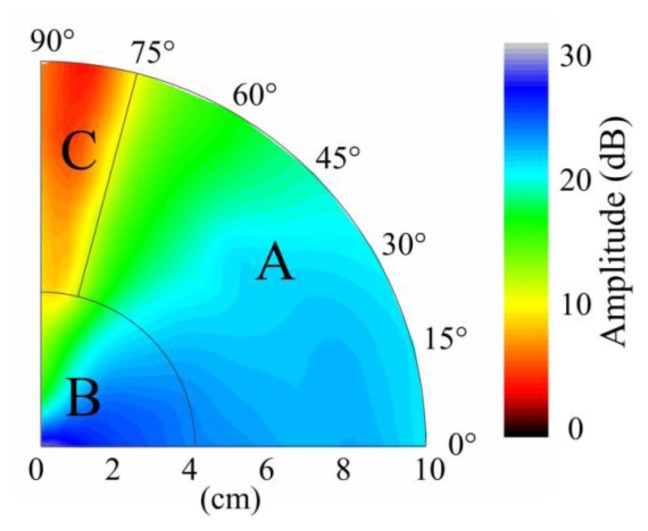
Sensitivity distribution properties of PS-FBG on an aluminum plate shown in logarithmic scale.

**Figure 4.**
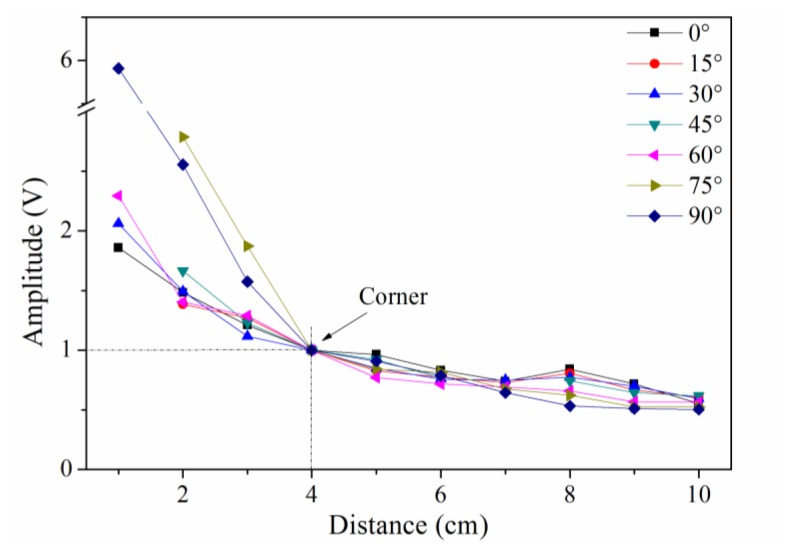
Amplitudes of detected waveforms at different distances for different angles after normalizing the detected waveforms' amplitudes to 4 cm.

Typically, the sensitivity is defined by the signal amplitude caused by a given level of input (ultrasound signal in this case). In this study, we use signal-to-noise ratio to reflect the sensitivity distribution of the sensor because the ultrasonic signal has the same original input voltage. Because the noise level, determined by the demodulation system, was constant in this experiment, the amplitudes of the detected waveforms can be used to directly evaluate the sensitivity of PS-FBG. A Hilbert transform was used to obtain the envelopes of the detected waveforms, and the amplitudes were then obtained by averaging the thrice-measured data. Figure 3 shows the sensitivity distribution properties of the PS-FBG on an aluminum plate in logarithmic scale. According to Figure 3, the detected waves' amplitudes decrease with the increase in distance between the PS-FBG sensor and the ultrasonic source. However, when the distance is smaller than 4 cm, the amplitudes are relatively large. Figure 4 was obtained by normalizing the amplitudes at 4 cm for different angles. In Figure 4, there is a clear corner at 4 cm. After 4 cm, the slopes of all curves are similar, due to attenuation of the ultrasonic wave. However, before 4 cm, the curves' slopes change greatly, especially in data from large angles, such as 75° and 90°. When the distance between sensor and actuator is small (corresponding to area B), the dynamic strain from the Y-axis caused by acceleration is relatively large and effectively shifts the Bragg wavelength, which leads to relatively large amplitudes.

**Figure 5.**
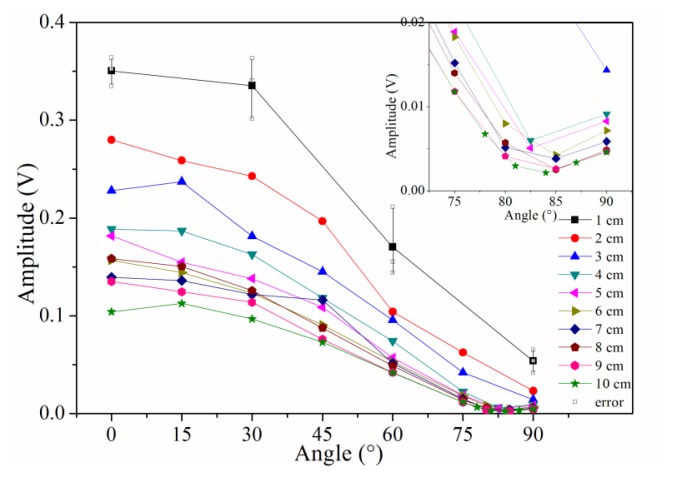
Amplitudes of detected waveforms to the angle change for different distances.

According to Figure 3, unlike a traditional PZT sensor (which is omnidirectionally sensitive), the sensitivity of the PS-FBG has an obvious directional dependence. Extracting the amplitudes at every distance in Figure 3, the sensitivity distribution at different angles is shown in Figure 5. As this figure depicts, the amplitudes generally decrease with the angle's increment. However, odd phenomena are observed from 75° to 90° after 4 cm, as amplified in the inset of Figure 5. These phenomena result because the waveforms with smallest amplitudes to the angle change are located around 84° rather than 90°, corresponding to area C in Figure 1b.

### 4.2. Phenomena in Area A

**Figure 6.**
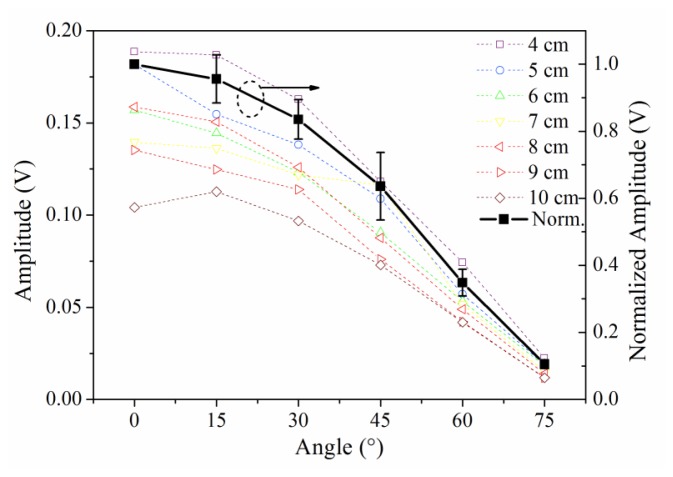
After normalization, the sensitivity and the angle show cosine relation in area A.

The sensitivity distribution in area A is simple because the effect from X-axis strain can be omitted due to the small value of *C_x_*, while the effect from Y-axis dynamic strain can also be omitted due to the plate's geometric dimensions. The open marks in Figure 6 are the data from area A. The black curve in Figure 6 is obtained by normalizing the data to the value in 0° according to different distances, and this figure shows the cosine relation between sensitivity and angle. Furthermore, in area A, the detected waveforms and the waves' initial phases are always constant for different distances.

### 4.3. Phenomena in Area B

**Figure 7.**
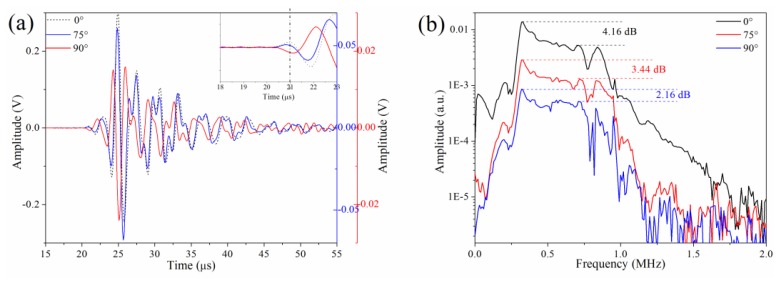
(**a**) Waveforms and (**b**) corresponding spectra of $D_{2}^{0}$, $D_{2}^{75}$ and $D_{2}^{90}$.

Figure 7a shows the detected waveforms at dots $D_{2}^{0}$, $D_{2}^{75}$ and $D_{2}^{90}$. The figure indicates that the amplitude of the wave at $D_{2}^{90}$ further decreases from $D_{2}^{75}$ and $D_{2}^{0}$. Additionally, the initial phase of the waveform from $D_{2}^{90}$ is opposite to the initial phase of $D_{2}^{0}$ or $D_{2}^{75}$, as shown in the inset in Figure 7a. This phenomenon matches the description in Section 3.1, in which lateral strain is dominant for $D_{2}^{90}$. Furthermore, the waveforms for $D_{2}^{90}$ and $D_{2}^{75}$ differ, and this effect results from different responses to certain frequencies when the ultrasonic wave couples to the fiber from different angles, as explained in Section 3.4. Figure 7b was obtained by performing a Fast Fourier Transform to corresponding waveforms. Although the corresponding spectra of $D_{2}^{0}$, $D_{2}^{75}$ and $D_{2}^{90}$ have a similar frequency range and shape, the responses to different frequencies are dissimilar. For example, the difference in amplitudes at frequency 0.32 MHz and 0.71 MHz is 4.16 dB at $D_{2}^{0}$. However, the amplitude difference decreases to 3.44 dB at $D_{2}^{75}$, while this difference further decreases to 2.16 dB at $D_{2}^{90}$. This decrease is a common phenomenon, which we can observe in almost all cases when the angle changes from 90° to 0°.

Moreover, no *α_min_* is observed. The absence of *α_min_* occurs because, in area B, the strains from all three axes are present, and the distance between PS-FBG and the ultrasonic source is very short; therefore, in this range, relatively large waveforms can always be detected.

### 4.4. Phenomena in Area C

**Figure 8.**
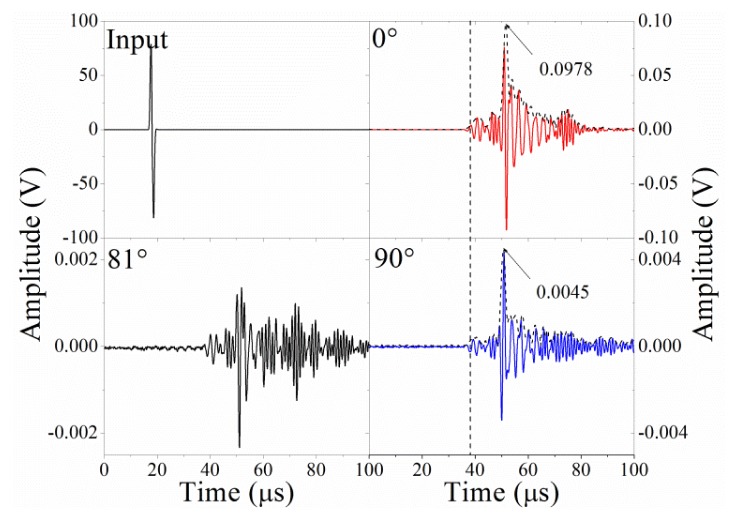
Waveforms of input signal, $D_{10}^{0}$, $D_{10}^{81}$ and $D_{10}^{90}$.

Figure 8 shows the input waveform and the detected waveforms for $D_{2}^{0}$, $D_{10}^{81}$ and $D_{10}^{90}$, respectively. The *α_min_* is around 84°, as shown in the inset of Figure 5, and this value can be explained by the formula for angle *α_min_*, as discussed in Section 3.3. After undergoing a Hilbert transform, the amplitudes of $D_{10}^{0}$ and $D_{10}^{90}$ are 0.0978 V and 0.0045 V, respectively, as shown in Figure 8. Substituting these values into Equation (9), the predicted angle where the minimal amplitude occurs is 87°, which is very close to the observed result.

**Figure 9.**
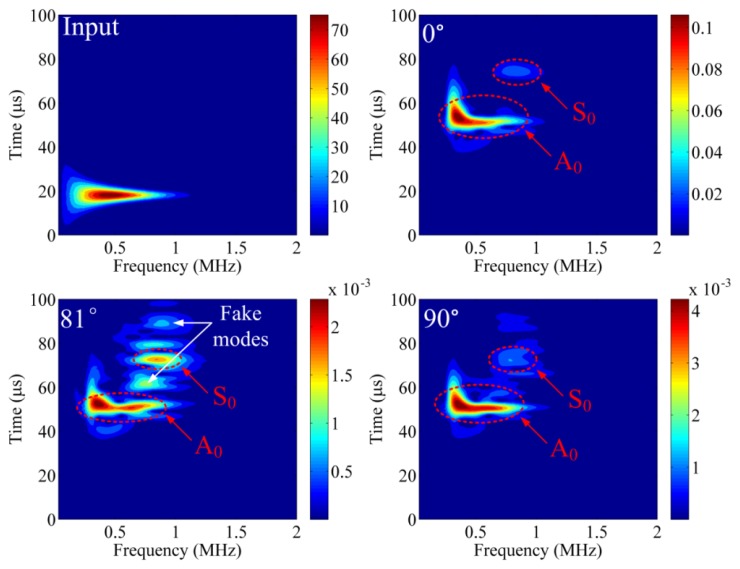
CWT results for input signal, $D_{10}^{0}$, $D_{10}^{81}$ and $D_{10}^{90}$.

Furthermore, in Figure 8, the waveform at $D_{10}^{90}$ is similar to the waveform at $D_{10}^{0}$ but has opposite phase, the reason for which is explained in Section 3.1. However, at the other observational dots between 75° and 90°, the waveforms are not constant and instead show complex waveform changes, such as the waveform for $D_{10}^{81}$. Continuous wavelet transforms (CWT) are used to further analyze the detected waveforms, as shown in Figure 9. In the CWT result for the input waveform, the frequency range approaches 1 MHz, and the input time is about 20 μs. The CWT results at $D_{10}^{0}$ and $D_{10}^{90}$ are very similar, and these results present a clear S_0_ mode and A_0_ mode. The S_0_ mode and A_0_ mode were determined by subtracting the input signal's arrival time and comparing with the theoretical dispersion curve for this aluminum plate. However, the A_0_ mode detected at $D_{10}^{90}$ contains slightly more relative energy in the high-frequency range. Also, the S_0_ mode for $D_{10}^{90}$ is more apparent than the S_0_ mode at $D_{10}^{0}$. These phenomena also can be explained as different responses to different frequencies, as discussed in Section 3.4. For example, S_0_ mode contains relative high frequency about 0.8 MHz, *i.e.*, it has relative short wavelength. When this component of wave propagates in Z axis, it does not effectively shift the Bragg wavelength compared to the case of static strain. However, when this component of wave propagates in the X or Y axis, it shifts the Bragg wavelength as the behavior of static strain because it is under quasi-uniform strain due to the short diameter of the fiber. Alternately, although the complex CWT result for $D_{10}^{81}$ contains a major A_0_ mode and S_0_ mode, there also exist several vague areas. These vague areas will confuse researchers and lead to analysis mistakes. Thus, we call these areas “fake modes” in Figure 9. The fake modes occur when the strain from Z axis and X/Y axis are approximately equivalent.

## 5. Discussion

For convenience, the sensitivity distribution properties of PS-FBG are divided into areas A, B, and C. However, the stain caused by ultrasonic waves in the plate is complex and defies simplistic divisions. For example, in area A, although the dominant strain is in the direction of the Z-axis, strains along the X- and Y-axes are also present. Similarly, in area B and area C, strains from all three orthogonal axes also exist. Thus, the above analysis does not fully encompass actual conditions. However, the analyses for results are sufficient to estimate the phenomena in each area.

Although the above discussion is based on results obtained from a PS-FBG sensor, the theory and phenomena can be extended to other FBG sensors or even optical fiber sensors because the PS-FBG or normal FBG is manufactured based on normal single-mode fiber, which has identical physical properties and responds similarly to ultrasonic waves. However, at high frequencies, the response of normal FBG should undergo additional consideration due to the relatively long effective grating length of about 2–3 cm when the ultrasonic wave propagates in the Z-axis. Furthermore, because of the high sensitivity achieved by PS-FBG and the balanced demodulation technique employed, we can observe small changes from 75° to 90° in area C; nevertheless, observation of these phenomena, such as *α_min_*, by normal FBG sensors may be difficult.

In this experiment, the fiber was glued to the surface of a plate. The other common attachment method is to embed the fiber into materials, such as carbon-fiber-reinforced plastic laminate. In this case, the coupling performance in the X-axis can be greatly enhanced. Thus, there is a possibility that the ultrasonic wave in the X-axis can also be clearly detected, which leads to a larger *α_min_*.

## 6. Conclusions

Based on experimental results obtained from 82 excitation dots, the sensitivity distribution properties of the PS-FBG sensor to ultrasonic waves (employing the surface attachment method) were identified and discussed. Although the strain from both the axial direction and the lateral direction can shift the Bragg wavelength of the PS-FBG, the amount and direction of this shift differ, which leads to the distance dependence and directional dependence of the sensitivity. Furthermore, the location of the ultrasonic source also influences the signals' initial phases, spectra, and CWT results. Moreover, other factors, including coupling performance, the attachment method, and the material's geometric dimensions, also influence the results. These results closely matched the theory. Because of identical physical properties for a single-mode fiber, the grating's sensitivity distribution properties can extend to a normal FBG or even an optical fiber sensor. These results can help optimize sensor networks and signal analyses in the fields of NDT and SHM.
